# A new acid isolated from *V. negundo* L. inhibits NLRP3 inflammasome activation and protects against inflammatory diseases

**DOI:** 10.3389/fimmu.2023.1174463

**Published:** 2023-04-20

**Authors:** Qianqian Di, Xibao Zhao, Jing Lin, Xunwei Li, Xiaoli Li, Haimei Tang, Ruihan Zhang, Weilie Xiao, Weilin Chen

**Affiliations:** ^1^Guangdong Provincial Key Laboratory for Regional Immunity and Diseases, Institute of Biological Therapy, Department of Immunology, Shenzhen University Medical School, Shenzhen University, Shenzhen, Guangdong, China; ^2^Key Laboratory of Medicinal Chemistry for Natural Resource, Ministry of Education, Yunnan Characteristic Plant Extraction Laboratory, Yunnan Provincial Center for Research & Development of Natural Products, State Key Laboratory for Conservation and Utilization of Bio-Resources in Yunnan, School of Pharmacy and School of Chemical Science and Technology, Yunnan University, Kunming, China

**Keywords:** Vitenegu acid, NLRP3, inflammasome, sepsis, peritonitis

## Abstract

The NLRP3 inflammasome plays a critical role in the innate immune response, and its excessive activation will cause pyroptotic cell death and be associated with the onset of inflammatory diseases. However, NLRP3 inflammasome targeting therapies are still to be implemented in the clinic setting. Here, we first isolated, purified and characterized a novel Vitenegu acid from *V. negundo* L. herb that specifically inhibits NLRP3 inflammasome activation, without affecting NLRC4 or AIM2 inflammasomes. Vitenegu acid blocks the oligomerization of NLRP3, thus inhibiting NLRP3 inflammasome assembly and activation. *In vivo* data show that Vitenegu acid exerts therapeutic effects on NLRP3 inflammasome-dependent inflammation. Taken together, our results suggest that Vitenegu acid is a candidate therapeutic agent for treating NLRP3 inflammasome related diseases.

## Graphical Abstract

Graphic Abstract

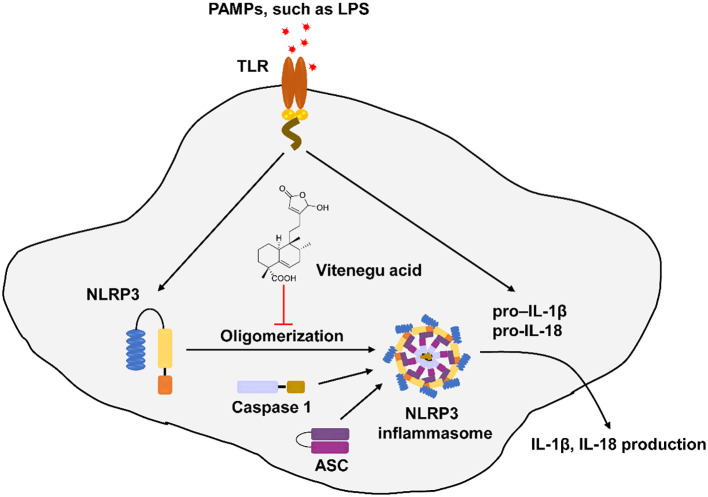

## 1 Introduction

NLRP3 inflammasome is a multiprotein complex composed by NLRP3 (NACHT, LRR and PYD domains-containing protein 3), ASC (Apoptosis-associated speck-like protein containing a CARD) and CASP-1 (Caspase-1) that modulates innate immune response by regulating the maturation of CASP-1 and cytokine processing, such as pro-IL-1β and IL-18 into their mature forms (1, 2). The NLRP3 inflammasome is activated by factors derived from pathogens associated molecular patterns (PAMPs) and endogenous damage associated molecular patterns (DAMPs) of host cells such as nigericin, ATP, amyloid β and monosodium urate crystals (3–5). Although NLRP3 inflammasome activation is key in immune response, its constitutive activation is deleterious in cells, and is associated with the onset of inflammation and related diseases, including type-2 diabetes, atherosclerosis, gout and Alzheimer’s disease (6–8).

Different host endogenous metabolites, such as dopamine and bile acids have been reported to inhibit NLRP3 inflammasome activation (9, 10). Additionally, distinct small molecules, such as MCC950, CY-09, ticagrelor, and tranilast have been shown to effectively inhibit the activity of NLRP3 inflammasome (11–14). Among these inhibitors, MCC950 is the best characterized inhibitor of NLRP3 and had been tested to treat rheumatoid arthritis in phase II clinical trials but failed because of hepatic toxicity (15). However, although these compounds had been showed potent therapeutic effect on mice model of NLRP3 inflammasome related diseases, the safety and underling mechanism remain to be elucidated. Besides, there is no specific inhibitor for NLRP3 in clinical use, so it is urgently needed to develop safe and effective inhibitors for the treatment of NLRP3-related inflammatory diseases.

Several herbs used in traditional Chinese medicine have shown anti-inflammatory activity and to ameliorate certain inflammatory conditions, such as *Premna szemaoensis* and *Callicarpa integerrima Champ.* (16, 17). Certain compounds extracted from these herbs, for example, oridonin from *Rabdosia rubescens* and Cryptotanshinone from *Salvia miltiorrhiza Bunge* are reported to inhibit NLRP3 inflammasome (18, 19). Therefore, plant-derived extracts might be potential alternative sources of safe and effective NLRP3 inflammasome specific.

*V. negundo* L. is a plant used in Chinese traditional medicine with anti-inflammatory and antinociceptive functions, but its underlying mechanism was unclear. Here we isolated and purified natural compounds from *V. negundo* L. and screened NLRP3 inflammasome inhibitors in the generated compound library. We found a novel Vitenegu acid from *V. negundo* L. herb that specifically inhibits the activation of NLRP3 inflammasome, without affecting NLRC4 or AIM2 inflammasomes. Vitenegu acid blocks the NLRP3 oligomerization and subsequently, NLRP3 inflammasome activation. Furthermore, our *in vivo* data show that Vitenegu acid acts as a therapeutic compound against NLRP3 inflammasome-dependent inflammation, including in LPS-induced and Alum-induced peritoneal inflammation. Together, our results point to Vitenegu acid as a candidate therapeutic agent in the context of NLRP3 inflammasome related diseases.

## 2 Materials and methods

### 2.1 Extraction and isolation of natural compounds

The air-dried acrial part of *V. negundo L.* (11.1kg) were extracted with 90% MeOH (3 × 4h). The MeOH extract was suspended in H_2_O and extracted with EtOAc at room temperature, yielding fraction of EtOAc (528.4 g) after removal of the solvent. The EtOAc fraction was subjected to silica gel column chromatography (CC), using gradient elution of CH_2_Cl_2_/Acetone (100:0, 50:1, 20:1, 10:1, 4:1, 7:3, 1:1, 0:100), to yield three major fractions (Fr.1~Fr.3) by their thin layer chromatograph (TLC) profiles. Fr.2 was submitted to MCI gel CHP-20P (75-150μm) in the function of gradient elution of MeOH/H_2_O (3:7, 1:1, 4:1, 9:1, 100:0) in order to remove pigment. And it was separated on RP-18 gel CC (5 cm × 45 cm), with a continuous gradient elution of MeOH/H_2_O (1:4 to 100:0), to obtain Fr.2.1~Fr.2.3. Fr.2.2 was applied to Sephadex LH-20 CC, eluting with CH_2_Cl_2_/MeOH(1:1), to afford three subfractions (Fr.2.2.1~Fr.2.2.3) by profiles of their TLC. Fr.2.2.2 was performed with a step gradient of n-hexane/EtOAc (10:1 to 0:1) which was divided into ten parts (Fr.2.2.2.1~Fr.2.2.2.10). Fr.2.2.2.7 was separated by semipreparative HPLC to afford Vitenegu acid (20.7mg) eluting with CH_3_CN/H_2_O (56:44).

### 2.2 Mice

Wild type C57BL6 mice (8 weeks, female) were purchased from Vital River Company (Beijing, China). Mice were specific pathogen-free and maintained under 12-hr light/dark cycle at 22-24°C and relative humidity 55 ± 5% with unrestricted access to food and water for the duration of the experiments. According to National Institutes of Health guidelines, all animal studies were approved by Shenzhen University School of Medicine Committees on Use and Care of Animals.

### 2.3 Reagents

ATP, nigericin, LPS, flagellin and poly(deoxyadenylic-deoxythymidylic) acid sodium salt (poly (dA:dT)) were purchased from InvivoGen (San Diego, USA). DMSO and MG132 was obtained from Sigma-Aldrich (Munich, Germany). MCC950 was purchased from Selleck (Houston, USA). Anti-DYKDDDDK-Tag antibody was purchased from MBL (Beijing, China). Anti-Myc-Tag, anti-HA-Tag and anti-β-actin were purchased from Proteintech (Wuhan, China). Anti-NLRP3, anti-ASC, anti-caspase-1 were obtained from AdipoGen (San Diego, USA). Anti-IL-1β and anti-NEK7 were purchased from Cell Signaling Technology (Danvers, USA). Secondary HRP-conjugated antibodies used were anti-mouse IgG, anti-rabbit IgG (Cell Signaling Technology, Danvers, USA).

### 2.4 Cell preparation and stimulation

Mice were injected intraperitoneally with 2 mL 3% fluid thioglycolate medium (Merck, Germany) for 4 days to obtain peritoneal macrophages. Mouse peritoneal macrophages were seeded at 1×10^6^ cells per hole in 12-well plates overnight. Then the cells were pretreated with Vitenegu acid or DMSO for 2 h and stimulated with LPS for 4 h. Next, the cells were changed to Opti-MEM (Invitrogen, Carlsbad, USA) and activated with NLRP3 inflammasome activator, nigericin (10 μM) for 45 min, ATP (2.5 mM) for 45 min, Alum (300 μg/mL, Thermo Fisher Scientific, Waltham, USA) for 3 h, or transfected with NLRC4 inflammasome activator Flagellin (0.5 μg/mL, InvivoGen, San Diego, USA) for 1 h, AIM2 inflammasome activator poly(dA:dT) (1 μg/mL, InvivoGen, San Diego, USA) for 1 h. Supernatants and cell lysates were collected and analyzed by ELISA or Western Blot.

### 2.5 Cell viability

To detect the viability of cells, the MTT assay was performed. Mouse peritoneal macrophages were seeded at 1×10^5^ cells in a 96-well plate, and incubated overnight at 37°C. Then, we treated the cells with different concentrations of Vitenegu acid (0-50 μM) for 12 h, followed by adding MTT reagent to the cell medium and incubating for 2 h, and cell residues were dissolved with 100 μL DMSO. Then, the optical density was measured at 590 nm using Microplate spectrophotometer.

### 2.6 ELISA

The concentration of IL-1β, IL-6 and TNFα in supernatants from serum, cell culture and peritoneal lavage fluid were assayed by ELISA kits (Invitrogen, Carlsbad, USA) according to the manufacturer’s instructions.

### 2.7 Immunoblot analysis

Immunoblot analysis was performed to validate the protein expressions as described previously (20).

### 2.8 Immunofluorescence

1×10^5^/mL mouse peritoneal macrophages were plated on coverslips in 12-well plates overnight. Then the medium was changed to Opti-MEM. The cells were pretreated with different concentration of Vitenegu acid for 2 h, and stimulated with LPS and nigericin as described. Next, the cells were washed three times with PBST for three times, fixed with 4% PFA for 30 min, and stained with ASC antibody and Alexa Fluor Plus 488 linked anti-Rabbit IgG (secondary antibody). Confocal microscopy analysis was carried out by using a Zeiss LSM 880.

### 2.9 ASC oligomerization

The mouse peritoneal macrophages were pretreated with different concentration of Vitenegu acid for 2 h, primed with 100 ng/mL LPS for 4 h, and stimulated with nigericin for 45 min. And the assay for ASC oligomerization were performed as described previously (10).

### 2.10 Alum-induced peritonitis mouse model

As adult male mice had thicker abdominal fat than the female mice, which would block the needle when acquiring peritoneal lavage fluids, so we used female mice to conduct our experiment. 8-week-old female mice were injected intraperitoneally with 10 mg/kg Vitenegu acid (dissolved in DMSO), and PBS containing 1 mg Alum was injected into the peritoneal cavity of mice. 10 h later, the mice were sacrificed. The peripheral blood, peritoneal lavage fluid and lung tissue were collected for subsequent analysis.

### 2.11 LPS-induced systemic inflammation *in vivo*

8-week-old C57BL6 mice were i.p. injected with Vitenegu acid (10 mg/kg) or DMSO, 30 min later, i.p. injected with LPS (20 mg/kg) for survival analysis. To induce *in vivo* cytokine secretion, mice were i.p. injected with Vitenegu acid (10 mg/kg) or DMSO, 30 min later, i.p. injected with LPS (10 mg/kg). After 4 h, the serum samples and peritoneal lavage fluids were collected and the cytokines were measured by ELISA.

### 2.12 Peritoneal lavage fluids acquisition and analysis

The peritoneal lavage fluids were wash with 1 mL of ice-cold PBS, and then centrifuged 500 g for 5 min to get cell-free supernatant. The concentration of IL-1β and TNFα in the peritoneal lavage fluid was detected using ELISA assay.

### 2.13 Flow cytometry assay

Cells collected from peritoneal lavage fluids was washed twice by cold PBS, and then stained with FITC-conjugated anti-CD11b (Biolegend, San Diego, CA, USA) and PE/Cy7-conjugated anti-Ly6G (Biolegend, San Diego, CA, USA) antibodies diluted in PBS with 2% FBS for 30 min at 4°C. The cells were washed two times with PBS/2% FBS. Finally, CD11b^+^ PECs and CD11b^+^ Ly6G^+^ neutrophils were analyzed by flow cytometer (CytoFLEX, Beckman Coulter, USA).

### 2.14 Molecular docking study

The crystal structure of NLRP3 (PDB ID: 6NPY) was used for molecular docking study by FlexX program (21, 22). Seventeen potential binding pockets were automatically detected by the Prepare Receptor module. The docking parameters remained as default.

### 2.15 Data and statistical analysis

The results in the text and figures are presented as mean ± SD of triplicate measurements in a representative experiment. The data was collected from three independent set of experiments. All statistical analysis was calculated using GraphPad Prism 6. Statistical significance of differences between two groups was analyzed by students t test. p<0.05 was considered as statistical significance of difference.

## 3 Results

### 3.1 Screening NLRP3 inflammasome inhibitors in *V. negundo.* L extract and cytotoxicity evaluation

To screening potent NLRP3 inflammasome inhibitors in *V. negundo. L* extract, we treated mouse peritoneal macrophages with compound from compounds library extracted in *V. negundo L.* 2 hours prior LPS exposure and nigericin activation. Nigericin is a pore-forming toxin which causes ion efflux and activates NLRP3 inflammasome (23). Then, the activated NLRP3 inflammasome will cleave pro-IL-1β and IL-18 and promote their secretion. Thus, we measured the secretion of IL-1β by ELISA assay. The results showed that Vitenegu acid significantly inhibited IL-1β secretion, indicating a potent impact in NLPR3 inflammasome activation (**Figure S1**). Besides, in comparation with other NLRP3 inflammasome inhibitors, Vitenegu acid also had similar or better inhibiting effect on nigericin induced IL-1β secretion at concentration 50 μM (**Figure S2**).

**Figure 1:**
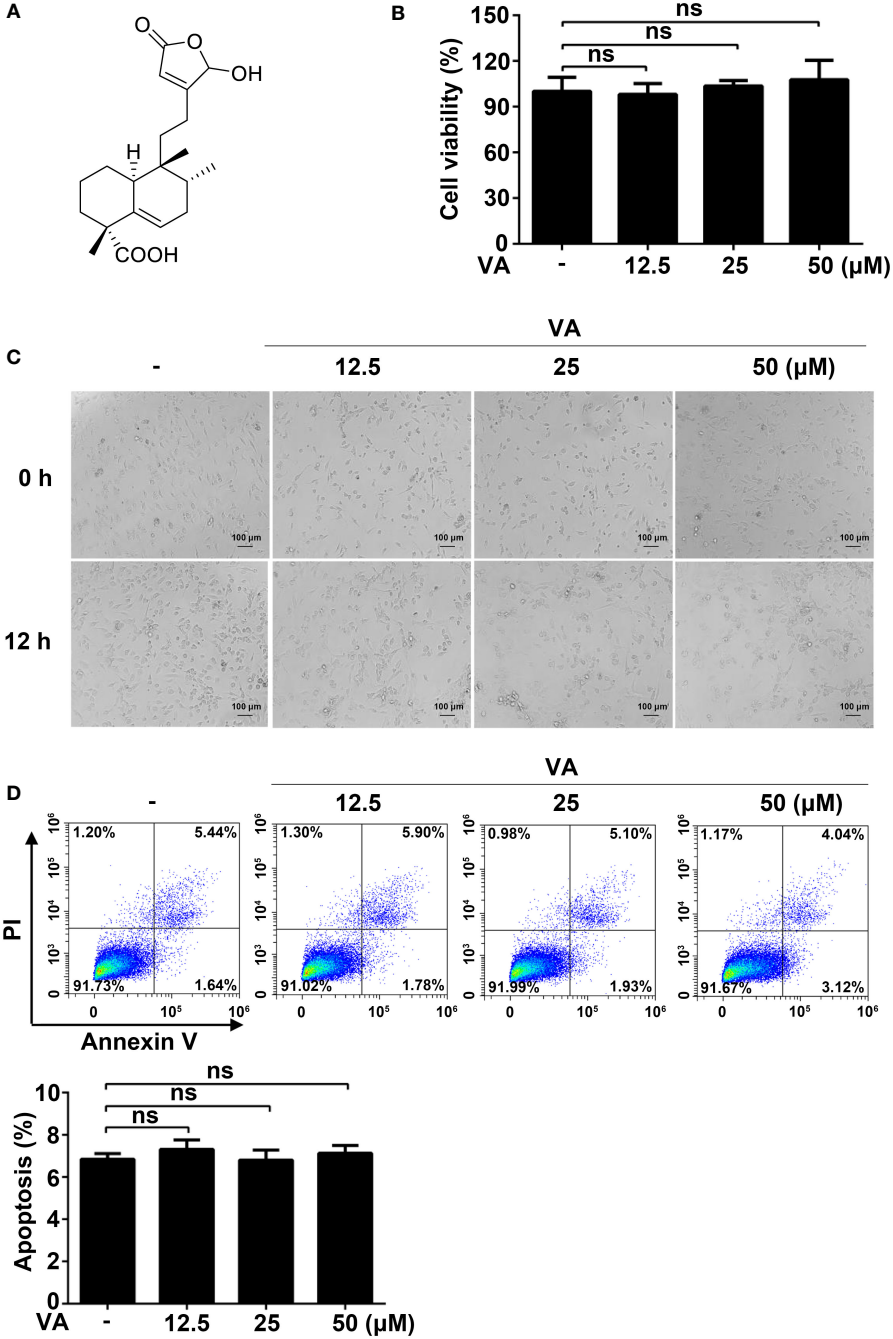
Screening NLPR3 inflammasome natural inhibitors from *V. negundo L*. **(A)** Chemical structure of Vitenegu acid. **(B)** Mouse peritoneal macrophages were treated with different concentrations of Vitenegu acid (0, 12.5, 25, 50 μM) for 12 h, and cell viability was evaluated by MTT assay. **(C)** Cell morphology was observed by optical microscopy (magnification 200×, scale bar 100 μm). **(D)** Cell viability was determined by flow cytometry staining with Annexin V-FITC/PI. The percentage of Annexin V positive cells was shown below. Values represent mean ± SD. The results from three independent experiments are presented as median with individual data. ns, not significant.

Vitenegu acid was isolated as a white powder, and its molecular formula was deduced as C_20_H_28_O_5_ by HRESIMS data at m/z 371.1826 [M+H]^+^ (calculated for C_20_H_29_O_3_, 317.1829), which suggested seven degrees of unsaturation (**Figure 1A**). The ^1^H NMR data contained two methyl singlets at *δ*_H_ 1.31 (3H, s, H-19), 0.86 (3H, overlap, H-20), one methyl doublet at *δ*_H_ 0.85 (3H, d, *J* = 6.8 Hz, H-17), two olefinic protons at *δ*_H_ 5.48 and *δ*_H_ 5.80 (H-12), and an oxymethine signal at *δ*_H_ 5.97 (1H, s) (**Figure S3**). The ^13^C NMR experiment indicated the presence of twenty carbon atoms, including three methyl groups at *δ*_C_ 14.7, 24.1, 22.1, two strongly deshielded *sp^2^* carbons at *δ*_C_ 171.4,172.0, two alkenyl carbons at *δ*_C_ 118.4, 116.9, a hemiacetal carbon at *δ*_C_ 99.4, and a carboxyl carbon at *δ*_C_ 182.6 (**Figure S4**). On the basis of HMQC and HMBC spectra, the existence of a *γ*-hydroxyl butenolide group was assumed from the presence of signals assignable to the hemiacetal carbon at *δ*_C_ 99.4 (C-16), two *sp^2^* carbons at *δ*_C_ 171.4 (C-13), 116.9 (C-14), and a carbonyl carbon at *δ*_C_ 172.0 (C-15) (**Figures S5**, **S6**). And the connection between the hexahydroindene skeleton and hydroxyl butenolide group was assisted by a two-atom aliphatic chain whose signals appeared as correlation peaks between H-11 and H-12 in ^1^H-^1^H COSY experiment and of H-20 with C-11 in HMBC spectrum. The relative configuration of Vitenegu acid was determined from ROESY (**Figures S7**-**11**). The ROESY correlations of H-10 with H-12/Me-17 deduced that H-10, H-11, H-12, and Me-17 were *α*-oriented. H-8 was assigned as *β*-oriented according to the correlation of H-20.

Before investigating the anti-inflammatory effects of Vitenegu acid, we first evaluated the cytotoxicity of Vitenegu acid. The impact of Vitenegu acid in cell viability was evaluated by MTT assay, in mouse peritoneal macrophages exposed to different concentrations of Vitenegu acid (0, 12.5, 25, 50 μM) for 12 h (**Figure 1B**). And the cell morphology was observed under the microscope (**Figure 1C**). The flow cytometry analysis also showed that the apoptosis of mouse peritoneal macrophages was not affected by up to 50 μM concentration of Vitenegu acid (**Figure 1D**). These results were showed that cell viability was not significantly affected by Vitenegu acid (0-50 μM), suggesting that its inhibitory effect on IL-1β secretion was not due to cell death.

### 3.2 Vitenegu acid inhibits NLRP3 inflammasome mediated inflammatory cytokines secretion

**Figure 2:**
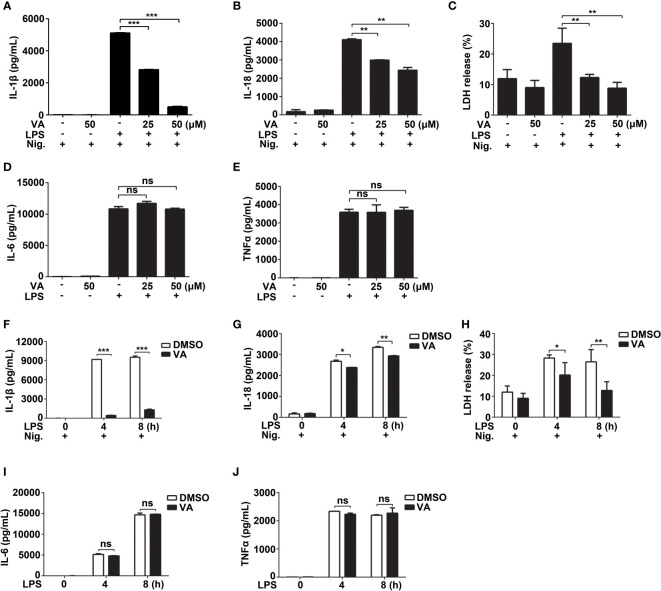
Vitenegu acid inhibits the release of IL-1β and IL-18 in mouse peritoneal macrophages. **(A–E)** Mouse peritoneal macrophages were pretreated with different concentrations of Vitenegu acid (0, 25, 50 μM) for 2 h, primed with LPS for 4 h and activated with nigericin for 45 min. Secretion of **(A)** IL-1β, **(B)** IL-18, **(C)** LDH, **(D)** IL-6 and **(E)** TNFα in the supernatants. **(F–J)** Mouse peritoneal macrophages were incubated with Vitenegu acid (50 μM) for 2 h, then primed with LPS for indicated hours and activated with nigericin for 45 min. Secretion of **(F)** IL-1β, **(G)** IL-18, **(H)** LDH, **(I)** IL-6 and **(J)** TNFα in the supernatants. Values represent mean ± SD. The results from at least three independent experiments. Significance is presented as *p<0.05, **p<0.01, ***p<0.001 versus the indicated group. ns, not significant.

As Vitenegu acid inhibited the release of IL-1β in the mouse peritoneal macrophages, we determined the half-maximal inhibitory concentration (IC_50_) of Vitenegu acid was approximately 28.6 μM (**Figure S12**). Besides, further studies also confirmed that Vitenegu acid inhibited nigericin-induced IL-1β and IL-18 secretion in a dose-dependent manner (**Figures 2A, B**). And the release of lactate dehydrogenase (LDH) induced by nigericin was blocked by Vitenegu acid (**Figure 2C**). In contrast, the production of IL-6 and TNFα was not affected (**Figures 2D, E**). In addition, the IL-1β, IL-18, IL-6 and TNFα mRNA expression was also not affected with Vitenegu acid pretreatment in mouse peritoneal macrophages plus LPS stimulation (**Figure S13**). To investigate the dynamic changes of inflammatory factors, we analyzed the effect of LPS stimulation for different time intervals up to 8 h. The data showed that Vitenegu acid significantly repressed the nigericin-induced secretion of IL-1β, IL-18 and LDH (**Figures 2F–H**). However, IL-6 and TNFα production was not affected (**Figures 2I, J**). As the mature and release of IL-1β and IL-18 were dependent on inflammasome mediated proteolysis carried out by the enzyme Caspase-1, while production of IL-6 and TNFα were independent of inflammasome. These results suggested that Vitenegu acid could inhibit the secretion of certain inflammatory cytokines whose release dependent on inflammasome, indicating Vitenegu acid had a potential inhibition on NLRP3 inflammasome activation.

### 3.3 Vitenegu acid specifically inhibits NLRP3 inflammasome activation

**Figure 3:**
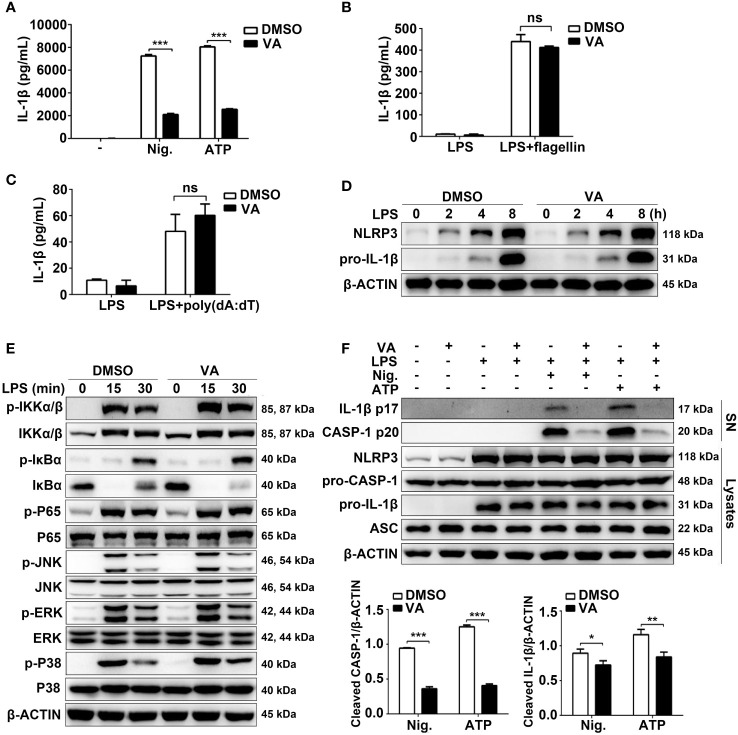
Vitenegu acid specifically inhibits NLRP3 inflammasome activation. **(A)** Mouse peritoneal macrophages were pretreated with Vitenegu acid or DMSO, and stimulated with LPS plus nigericin or ATP. ELISA assay was used to measure the secretion of IL-1β. **(B)** IL-1β in the supernatants of Vitenegu acid pretreated mouse peritoneal macrophages stimulated with LPS plus flagellin transfection. **(C)** IL-1β in the supernatants of Vitenegu acid pretreated mouse peritoneal macrophages stimulated with LPS and transfected with poly(dA:dT). **(D)** Western blot of cell lysates from Vitenegu acid pretreated mouse peritoneal macrophages stimulated with LPS. **(E)** Western blots analysis the expression of key protein in NF-κB and MAPK signaling pathway. **(F)** Western blot of cell lysates and supernatant from Vitenegu acid pretreated mouse peritoneal macrophages stimulated with LPS plus nigericin or ATP. (Values in **(A–C)** and **(F)** are expressed as the mean ± SD of three independent experiments. **(D–F)** are representative of three independent experiments. Significance is presented as *p<0.05, **p<0.01, ***p<0.001 versus the indicated group. ns, not significant.

To determine whether Vitenegu acid was a common inhibitor for NLRP3 inflammasome, we examined other NLRP3 agonists. ATP can be recognized by the purinergic P2X7 receptor and induce potassium efflux (24). Similar to nigericin, pretreatment with Vitenegu acid also inhibited ATP-induced IL-1β secretion (**Figure 3A**). We also tested whether Vitenegu acid could inhibit other inflammasomes, such as NLRC4 or AIM2 inflammasome activation. The results showed that Vitenegu acid had no effect on NLRC4 or AIM2 inflammasome activation, which were triggered by flagellin or poly(dA:dT) transfection, respectively (**Figures 3B, C**). To investigate the inhibition of Vitenegu acid on NLRP3 inflammasome, we detected the expression of NLRP3 and pro-IL-1β. The results showed that Vitenegu acid had no effect on NLRP3 and pro-IL-1β expression (**Figure 3D**). Moreover, Vitenegu acid treatment did not affect the transduction of NF-κB and MAPK signaling pathways in mouse peritoneal macrophages, which is essential for the expression of NLRP3 and pro-IL-1β (**Figure 3E**). However, Vitenegu acid treatment blocked nigericin or ATP -induced caspase-1 activation and IL-1β secretion in mouse peritoneal macrophages (**Figure 3F**). The results indicated that Vitenegu acid is a specific inhibitor for NLRP3 inflammasome activation, but had no impact on LPS-induced priming.

### 3.4 Vitenegu acid attenuates NLRP3 oligomerization and inflammasome assembly

**Figure 4:**
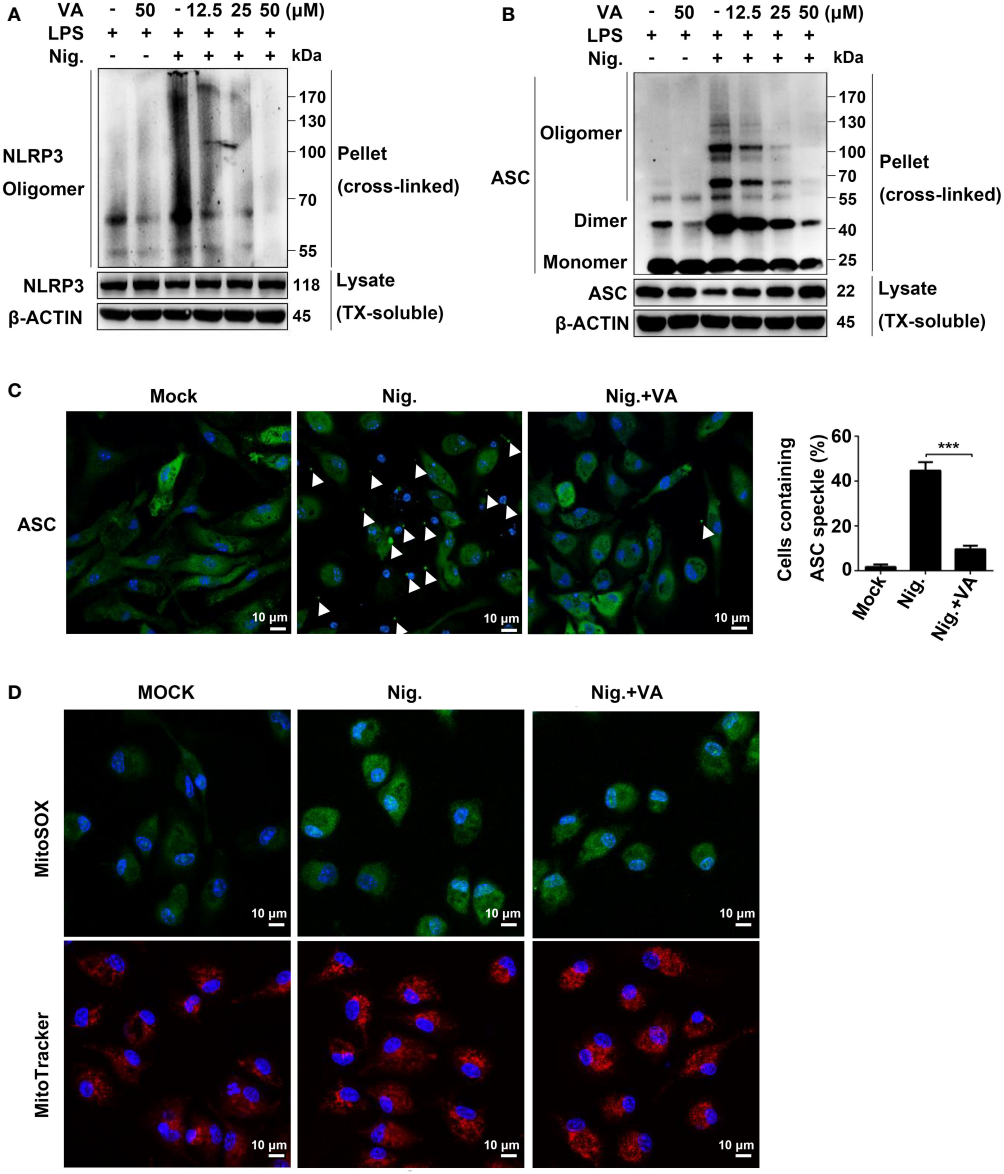
Vitenegu acid suppresses NLRP3 oligomerization and inflammasome assembly. **(A)** Western blot analysis of cross-linked NLRP3 in the NP-40-insoluble pellet of mouse peritoneal macrophages treated with different doses of Vitenegu acid, primed with LPS, and activated with nigericin. **(B)** Western blot analysis of cross-linked ASC in the NP-40-insoluble pellet mouse peritoneal macrophages treated with different doses of Vitenegu acid, primed with LPS, and activated with nigericin. **(C)** ASC speck formation in LPS and nigericin stimulated mouse peritoneal macrophages with or without Vitenegu acid. Quantification of ASC speck is shown at the right panel. **(D)** Confocal microscopy of LPS-primed mouse peritoneal macrophages treated with Vitenegu acid (50 μM) and stimulated with nigericin. Cells were stained with MitoSOX and MitoTracker Red. Nuclei were stained with DAPI (original magnification 630×, scale bar 10 μm). Significance is presented as ***p<0.001 versus the indicated group.

To determine whether Vitenegu acid inhibited the NLRP3 inflammasome assembly, we detected the oligomerization of NLRP3 in LPS plus nigericin-stimulated mouse peritoneal macrophages. In crosslinking experiments, Vitenegu acid could reduce the oligomerization of NLRP3 (**Figure 4A**). ASC oligomerization is critical for NLRP3 inflammasome assembly and the subsequent caspase-1 activation (25). In western blotting experiments performed with cross-linked insoluble cellular fraction, ASC oligomerization in the insoluble fraction of mouse peritoneal macrophages was markedly inhibited by Vitenegu acid pretreatment compared to DMSO controls (**Figure 4B**). During inflammasome activation, ASC forms branched fiber-like structures (26). We also confirmed that these ASC specks were inside of cells (**Figure S14**). High-resolution imaging of ASC specks in mouse peritoneal macrophages revealed a significant decrease in the number and size of ASC specks in Vitenegu acid pretreated cells compared to control, indicating that Vitenegu acid acts upstream of ASC oligomerization to inhibit the subsequent caspase-1 activation and IL-1β production (**Figure 4C**). Mitochondria damage, represented as mitochondria fission, clustering and reactive oxygen species production, is proposed as an upstream signaling event of NLRP3 activation (27). However, Vitenegu acid treatment had little effects on nigericin-induced mitochondrial damage and ROS production (**Figure 4D**), indicating that Vitenegu acid does not affect the signaling events of upstream of NLRP3 inflammasome formation.

### 3.5 Vitenegu acid inhibits the interaction of NLRP3-NLRP3

**Figure 5:**
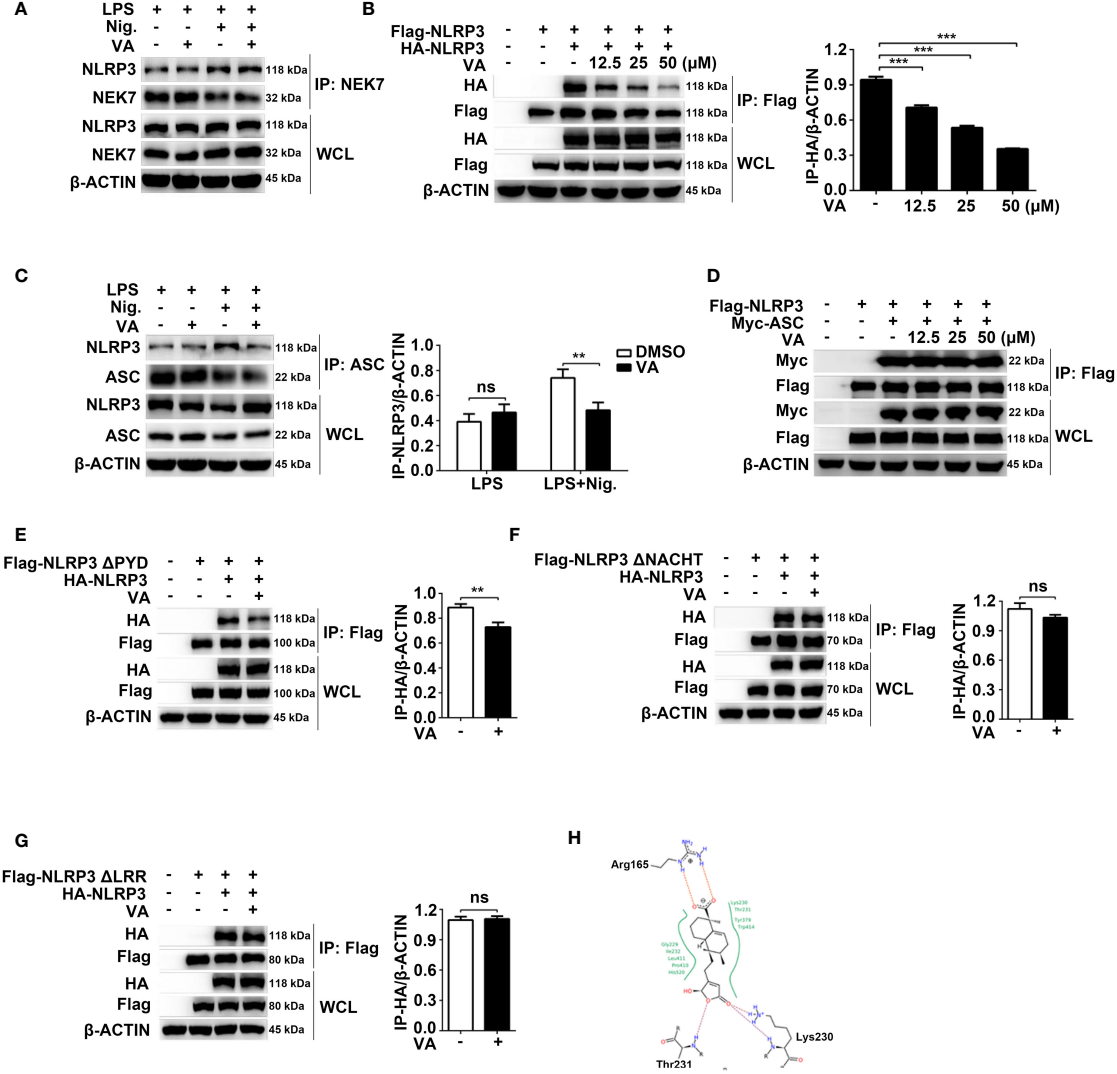
Vitenegu acid inhibits NLRP3 oligomerization. **(A)** Immunoprecipitation analysis of the endogenous interaction between NLRP3 and NEK7 in Vitenegu acid pretreated mouse peritoneal macrophages primed with LPS and activated by nigericin. **(B)** Immunoprecipitation and immunoblot analysis of the interaction between Flag-NLRP3 and HA-NLRP3 in the lysates from 293T cells incubated with different concentrations of Vitenegu acid. Quantification of HA-tagged NLRP3 from three independent experiments. **(C)** Immunoprecipitation analysis of endogenous interaction between NLRP3 and ASC in Vitenegu acid pretreated mouse peritoneal macrophages primed with LPS and activated by nigericin. **(D)** Immunoprecipitation analysis of the interaction between Flag-NLRP3 and Myc-ASC in the lysates from 293T cells incubated with different concentrations of Vitenegu acid. **(E–G)** Immunoprecipitation and immunoblot analysis in the lysates of 293T cells treated with Vitenegu acid (50 μM) or DMSO (1:1000), and quantification of HA-tagged NLRP3. Interaction between **(E)** HA-NLRP3 and Flag-NLRP3 ΔPYD; **(F)** HA-NLRP3 and Flag-NLRP3 ΔNACHT; **(G)** HA-NLRP3 and Flag-NLRP3 ΔLRR. **(H)** Predicted binding modes of Vitenegu acid with the NLRP3 protein. Significance is presented as **p<0.01, ***p<0.001 versus the indicated group. ns, not significant.

We then investigated the effects of Vitenegu acid on the formation NLRP3 inflammasome. One essential step for NLRP3 inflammasome assembly is the interaction between NEK7 and NLRP3, which is critical for the subsequent NLRP3 oligomerization and recruitment of ASC to NLRP3 (28, 29). Indeed, we observed that nigericin treatment induced endogenous NEK7-NLRP3 interaction in mouse peritoneal macrophages. However, the interaction was not affected by Vitenegu acid treatment (**Figure 5A**), indicating a downstream inhibitory effect of Vitenegu acid in NLRP3 inflammasome activation. NLRP3 oligomerization and recruitment of ASC to NLRP3 oligomers are essential steps for NLRP3 inflammasome activation (1, 8, 30). Thus, we investigated if Vitenegu acid prevents NLRP3-NLRP3 or NLRP3-ASC interactions. We overexpressed Flag-tagged NLRP3 and HA-tagged NLRP3 in 293T cells. The results suggested that Vitenegu acid blocks NLRP3-NLRP3 interaction in a dose-dependent manner (**Figure 5B**). Moreover, the nigericin-induced endogenous NLRP3-ASC interaction was dramatically inhibited by Vitenegu acid in mouse peritoneal macrophages (**Figure 5C**). However, Vitenegu acid did not affect ectogenic NLRP3-ASC interaction overexpressed in 293T cells, indicating that Vitenegu acid did not directly inhibit the interaction of NLRP3 and ASC (**Figure 5D**). These results indicated that Vitenegu acid prevented direct NLRP3-NLRP3 interactions, thus preventing NLRP3 oligomerization and ASC recruitment.

NLRP3 contains three domains: an N-terminal pyrin domain (PYD, 1-220 aa), a centrally located nucleotide-binding domain (NACHT, 220-389 aa), and a C-terminal leucine-rich repeat (LRR, 389-1037 aa). To explore potential Vitenegu acid binding domains, we used three NLRP3 truncated mutants: ΔPYD lacking the pyrin domain, ΔNACHT lacking the NACHT domain, and ΔLRR lacking the LRR domain. Coimmunoprecipitation experiments revealed that Vitenegu acid suppressed the interaction between NLRP3 ΔPYD and NLRP3 (**Figure 5E**), whereas the interaction between NLRP3 and NLRP3 ΔNACHT/ΔLRR were not affected (**Figures 5F, G**). To further investigate the interaction between Vitenegu acid and NLRP3, we performed a molecular docking study. There are 17 potential small molecule binding sites described in NLRP3. **Figure S15** shows the binding modes of the compound with the seven top-scored binding sites. The highest score (-27.86) was observed in the ADP binding site, where Vitenegu acid formed hydrogen bonds with the side chain of lysine 230 (Lys230) and the mainchain of Thr231, as well as a salt bridge with the side chain of arginine 165 (Arg165, **Figure 5H**). These findings reveal that Vitenegu acid inhibits the assembly of NLRP3 inflammasome by disturbing NLRP3-NLRP3 interactions.

### 3.6 Vitenegu acid mitigates LPS-induced systemic inflammation

**Figure 6:**
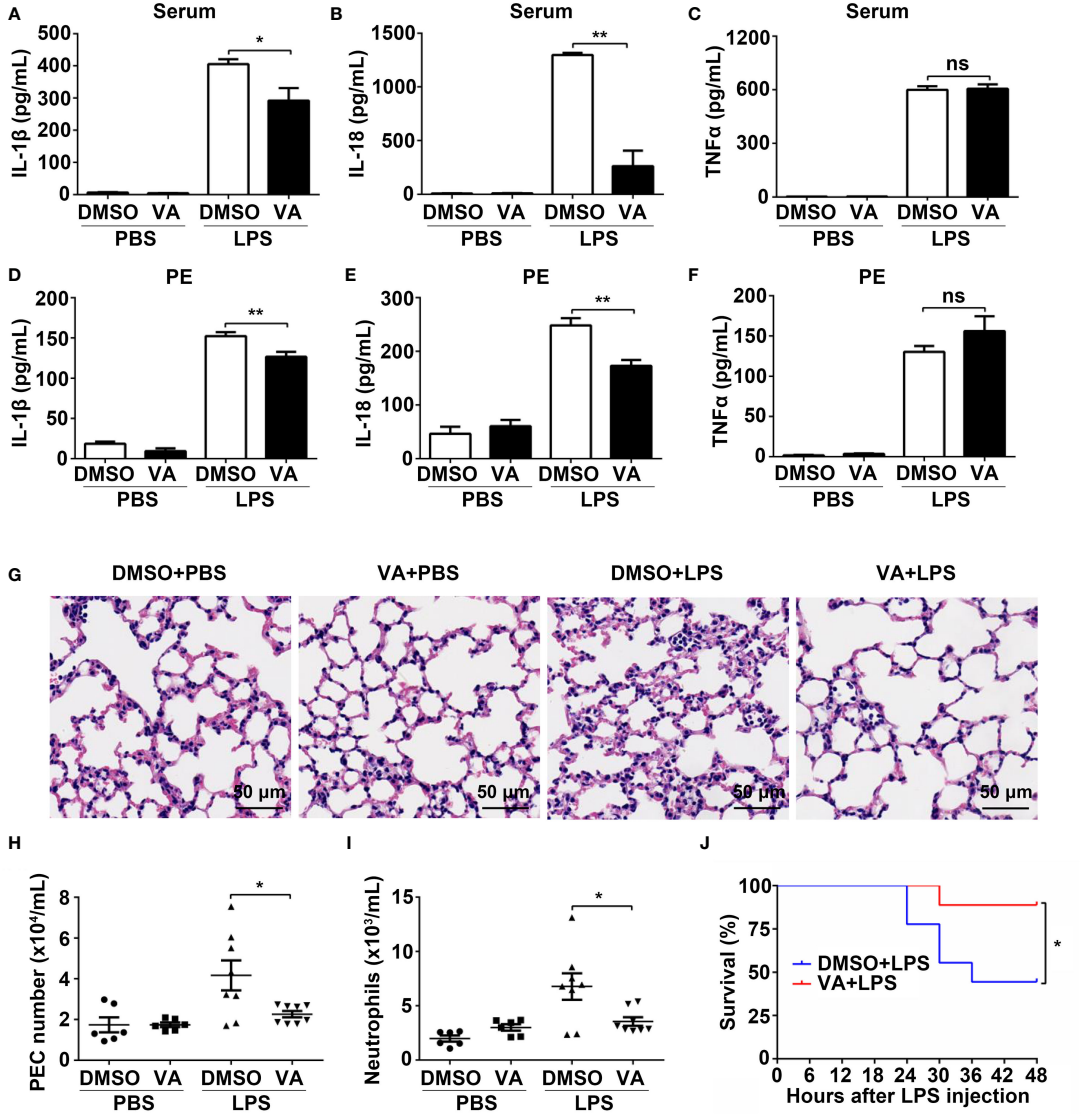
Vitenegu acid mitigates LPS-induced systemic inflammation. **(A–I)** 8-week-old C57BL6 mice (n=6 per group) were i.p. (intraperitoneal injection) injected with DMSO or Vitenegu acid (10 mg/kg). After 30 min, mice were i.p. injected with LPS (10 mg/kg). 4 hours later, Serum IL-1β **(A)**, IL-18 **(B)** and TNFα **(C)** were analyzed by ELISA. IL-1β **(D)**, IL-18 **(E)** and TNFα **(F)** levels in the lavage fluid were analyzed by ELISA. **(G)** H&E staining of left lung sections (magnification 400×, scale bar 50 μm). CD11b^+^ PECs **(H)** and CD11b^+^ Ly6G^+^ neutrophil quantification **(I)** was performed in the peritoneal lavage by flow cytometry. **(J)** Mice (n=9 per group) were i.p. injected with DMSO or Vitenegu acid (10 mg/kg). After 30 min, mice were i.p. injected with LPS (20 mg/kg). Survival was monitored for 48h Significance is presented as *p<0.05, **p<0.01 versus the indicated group. ns, not significant.

Following our previous observations on Vitenegu acid-mediated inhibition of NLRP3 inflammasome and reduction of IL-1β secretion *in vitro*, we next determined if Vitenegu acid could exert anti-inflammatory effects *in vivo*, using a mouse model for septic shock. The mice were intraperitoneally injected with Vitenegu acid (10 mg/kg) or equal volume of DMSO 30 min before LPS (10 mg/kg) challenge for 4 h. Firstly, we examined whether Vitenegu acid had potent toxic to mice. The H&E staining showed normal cell morphology in the main organs (liver, heart, lung, and kidney, **Figure S16A**), both the nuclei number per HPF and percentage of CD45^+^ cells in heart and kidney were also not affected (**Figures S16B–E**), indicating that Vitenegu acid had no toxic to mice at the treatment concentration. We then detected if Vitenegu acid could block the induction of pro-inflammatory cytokines and observed that it reduced the production of IL-1β and IL-18 in the serum, without affecting the production of TNFα (**Figures 6A–C**). Similar results were obtained in the peritoneal lavage fluid (**Figures 6D–F**). H&E staining indicated that Vitenegu acid was able to suppress inflammatory cell infiltration and lung injury (**Figure 6G**). In addition, Flow cytometry analysis showed a significant reduction in the number of total CD11b^+^ peritoneal exudate cells (PECs) and CD11b^+^ Ly6G^+^ neutrophils recruited in the enterocoelia, in Vitenegu acid pre-treated mice (**Figures 6H, I**). Then we examined the effect of Vitenegu acid on the survival rate of mice with lethal LPS-induced septic shock. The results showed a reduction in mortality in LPS plus Vitenegu acid-treated animals, compared to LPS plus DMSO-treated counterparts (**Figure 6J**). Collectively, the results demonstrate that Vitenegu acid inhibits LPS-induced systemic inflammation *via* suppression of NLRP3 inflammasome.

### 3.7 Vitenegu acid has beneficial effects in mouse model of Alum induced peritonitis

**Figure 7:**
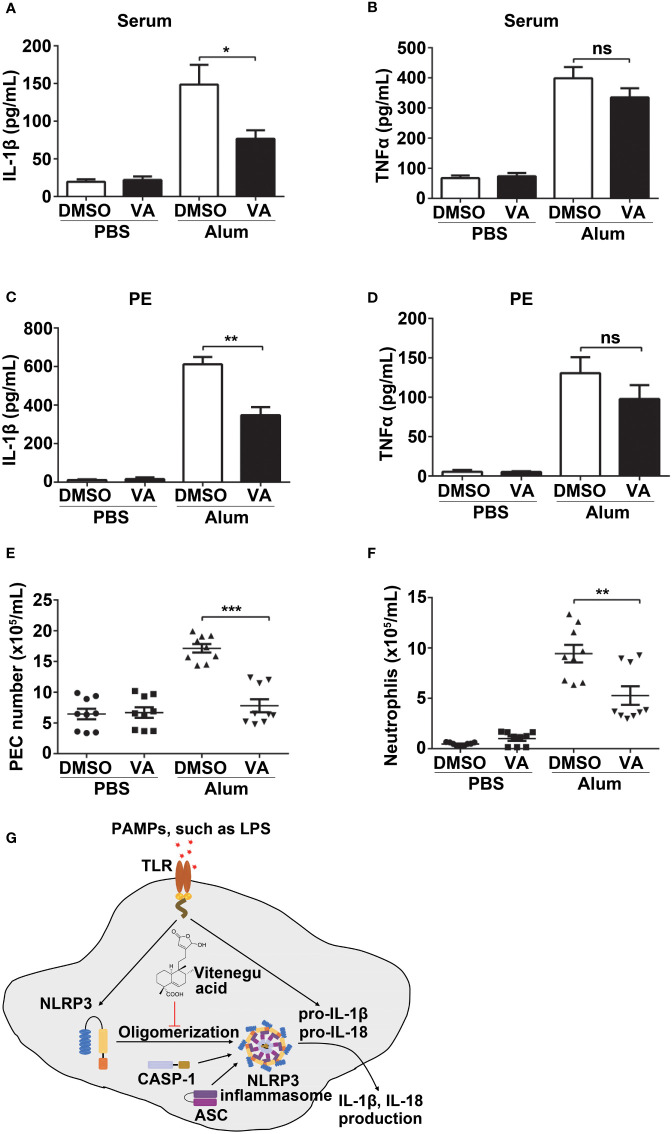
Vitenegu acid suppresses NLRP3-dependent peritonitis *in vivo*. 8-week-old C57BL/6 mice (n=8-9 per group) that were pretreated with Vitenegu acid 30 min before intraperitoneal injection of Alum (1 mg). 10 h later, ELISA quantification of serum IL-1β **(A)** and TNFα **(B)**. ELISA quantification of IL-1β **(C)** and TNFα **(D)** in the peritoneal cavities. **(E)** Flow cytometry quantification of total inflammatory cells (CD11b^+^ cells) in the peritoneal cavities. **(F)** Flow cytometry quantification of CD11b^+^ Ly6G^+^ neutrophils in the peritoneal cavities **(G)** Schematic representation of Vitenegu acid-mediated inhibition of NLRP3 inflammasome activation by interfering with NLRP3 aggregation. The data shown are mean ± SEM. Significance is presented as *p<0.05, **p<0.01, ***p<0.001 versus the indicated group. ns, not significant.

To further study the protective effect of Vitenegu acid in NLRP3 associated diseases, we next determined if Vitenegu acid can effectively inhibit NLRP3 inflammasome activation in a mouse peritonitis model. Mice pretreated with Vitenegu acid or DMSO were intraperitoneally injected with Alum to trigger peritonitis. As expected, Vitenegu acid also markedly reduced the serum levels of IL-1β but not the serum level of TNFα (**Figures 7A, B**). Moreover, IL-1β secretion in the lavage fluid was significantly decreased in Vitenegu acid pretreated mice, whereas the production of TNFα was not affected (**Figures 7C, D**). The recruitment of inflammatory cells in the peritoneal cavities were then analyzed by flow cytometry. The number of total CD11b^+^ PECs and CD11b^+^ Ly6G^+^ neutrophils recruited upon Alum challenge was markedly decreased by Vitenegu acid treatment (**Figures 7E, F**). Altogether, these results demonstrate that Vitenegu acid inhibits NLRP3 inflammasome activation and subsequent immune cell recruitment accumulation in mouse peritonitis.

## 4 Discussion

*V. negundo* L. herb is widely used in Chinese traditional medicine. *V. negundo L.* has been found to stimulate blood circulation, to counteract inflammation, to reduce swelling, and to support detoxification. However, its physiological and molecular mechanisms are still unclear. In this study, we report that Vitenegu acid extracted from the *V. negundo* L. herb specifically inhibits NLRP3 inflammasome activation, *in vitro* and *in vivo*, with beneficial effects against NLRP3-related inflammatory diseases, indicating that Vitenegu acid may be a safe candidate to treat NLRP3-related diseases (**Figure 7G**).

The activation of NLRP3 inflammasome requires two steps. First, the activation of Toll-like receptors by its ligands such as lipopolysaccharides, results in transcriptional activation of pro-inflammatory cytokines. Second, the activation of the inflammasome component, by the assembly NLRP3 inflammasome subunits, leading to caspase-1 activation, which in turn cleaves pro-cytokines into their active forms (31, 32). Our data showed that Vitenegu acid did not affect NF-κB and MAPK signaling pathway mediated pro-IL-1β and NLRP3 expression and TNFα production both in mouse peritoneal macrophages and serum of NLRP3-related diseases models, however, the mature of IL-1β and IL-18 were reduced in Vitenegu acid treated cells, which indicated that Vitenegu acid mainly inhibited the assembly and activation of NLRP3 inflammasome. Moreover, Vitenegu acid did not affect NLRC4 and AIM2 inflammasome activation, as observed from the unaltered IL-1β levels. Thus, we suggest that Vitenegu acid specifically inhibits NLRP3 inflammasome activation.

Activation of NLRP3 inflammasome, in response to external stimuli, is mediated by the interaction of NEK7 with NLRP3, which then promotes NLRP3 oligomerization (21, 33, 34). We observed that Vitenegu acid did not affect the interaction between NEK7 and NLRP3, indicating that Vitenegu acid targets downstream processes. In fact, we observed that Vitenegu acid inhibited the NLRP3-NLRP3 interaction. Further molecular docking experiments indicated that Vitenegu acid interacts with NLRP3 at its ADP binding site. However, the detailed combination mode and effect of Vitenegu acid-NLRP3 interaction remains to be explored.

In summary, our study demonstrates that Vitenegu acid inhibits NLRP3 inflammasome activation by suppressing NLRP3 oligomerization, thus blocking NLRP3 inflammasome assembly. Our results further demonstrate that Vitenegu acid alleviates LPS-induced systemic inflammation and Alum-induced peritonitis *in vivo*. Collectively, we propose that Vitenegu acid is a potential candidate for the treatment of NLRP3-related diseases.

## Data availability statement

The raw data supporting the conclusions of this article will be made available by the authors, without undue reservation.

## Ethics statement

The animal study was reviewed and approved by the Animal Care and Use Committee of Shenzhen University Medical School (Approval Number: A202201426).

## Author contributions

WC, WX and QD participated in the research design. QD, XZ, XL, HT, JL and RZ conducted the experiments. XZ, QD, WX and XL performed the data analysis. QD, WC, WX and RZ contributed to the writing of the manuscript. All authors contributed to the article and approved the submitted version.
