# Supplementary material for: A new acid isolated from V. negundo L. inhibits NLRP3 inflammasome activation and protects against inflammatory diseases
Source: Front Immunol. 2023 Apr 20;14:1174463. doi: 10.3389/fimmu.2023.1174463 (PMC10157029; doi:10.3389/fimmu.2023.1174463)
Supplement: Supplementary file 1 [file DataSheet_1.pdf]

Supplementary Material

A new acid isolated from *V. negundo* L. inhibits NLRP3 inflammasome activation and protects against inflammatory diseases

Qianqian Di [1, #], Xibao Zhao [1, #], Jing Lin [2, #], Xunwei Li [1], Xiaoli Li[2], Haimei Tang [1], Ruihan Zhang [2], Weilie Xiao [2, *], Weilin Chen [1, *]

[1] Guangdong Provincial Key Laboratory for Regional Immunity and Diseases, Institute of Biological Therapy, Department of Immunology, Shenzhen University Medical School, Shenzhen University, Shenzhen, Guangdong, China
[2] Key Laboratory of Medicinal Chemistry for Natural Resource, Ministry of Education, Yunnan Characteristic Plant Extraction Laboratory, Yunnan Provincial Center for Research & Development of Natural Products, State Key Laboratory for Conservation and Utilization of Bio-Resources in Yunnan, School of Pharmacy and School of Chemical Science and Technology, Yunnan University, Kunming, China
[#] These authors contributed equally to this paper.

*** Correspondence:**
Prof. Weilie Xiao, Tel: 0871-65223243. Fax: 0871-65223243. Email: xiaoweilie@ynu.edu.cn or_Prof. Weilin Chen, Tel: 0755-26910334. Fax: 0755-26910334. Email: cwl@szu.edu.cn

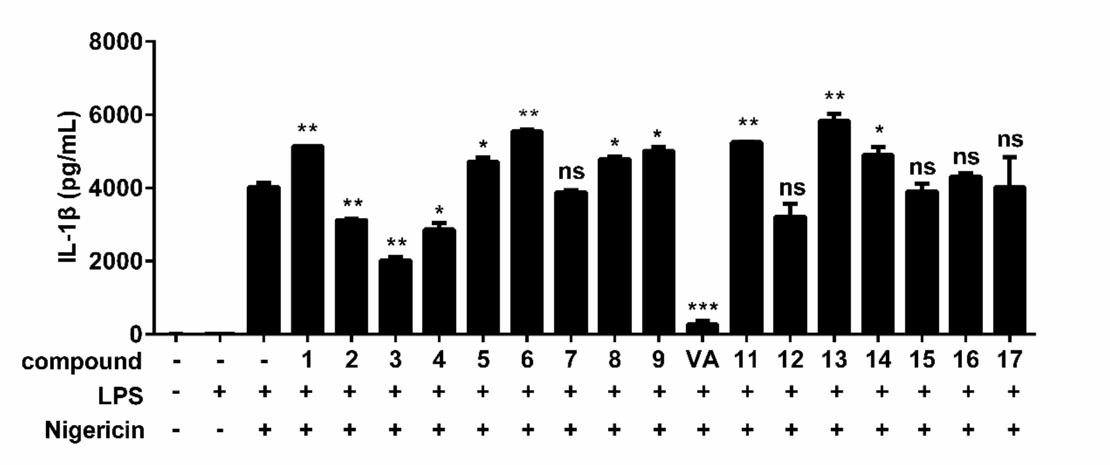

Figure S1. Screening potent NLRP3 inflammasome inhibitors from compounds library extracted in *V. negundo L.* ELISA analysis of IL-1β secretion in mouse peritoneal macrophages pretreated with DMSO or various compounds (50 μM each) for 2 h, then LPS (100 ng/mL) stimulation for 4 h and nigericin (5 μM) activation for 45 min. Significance is presented as *$p < 0.05$, **$p < 0.01$, ***$p < 0.001$ versus the DMSO pretreated group. ns, not significant.

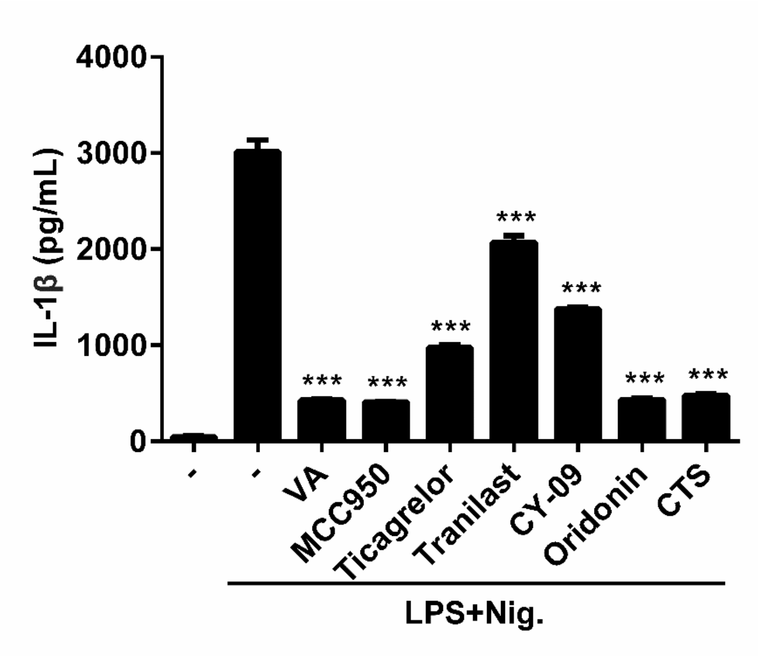

Figure S2. Comparison Vitenegu acid and other NLRP3 inflammasome inhibitors. ELISA analysis of IL-1β secretion in mouse peritoneal macrophages pretreated with Vitenegu acid (50 μM) or other NLRP3 inflammasome inhibitors (50 μM each) for 2 h, then LPS (100 ng/mL) stimulation for 4 h and nigericin (5 μM) activation for 45 min. Significance is presented as ***$p < 0.001$ versus the DMSO pretreated group.

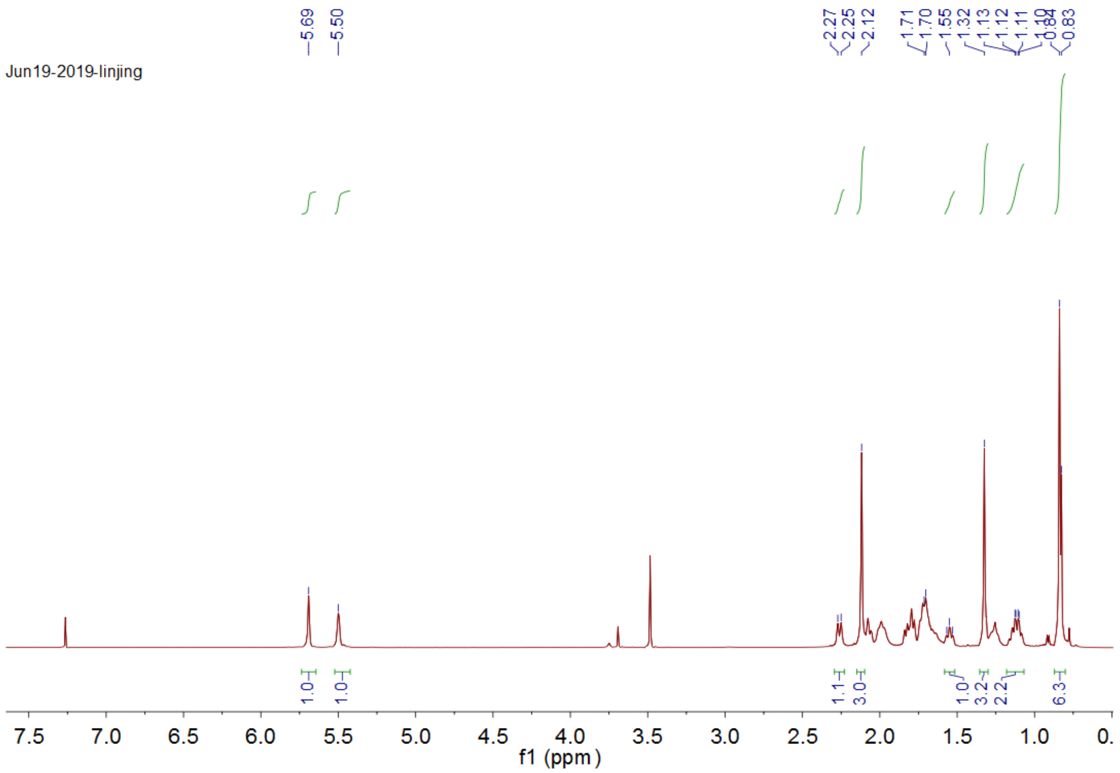

Figure S3. ^{1}H NMR spectrum of Vitenegu acid recorded in $CDCl_3$ at 600 MHz.

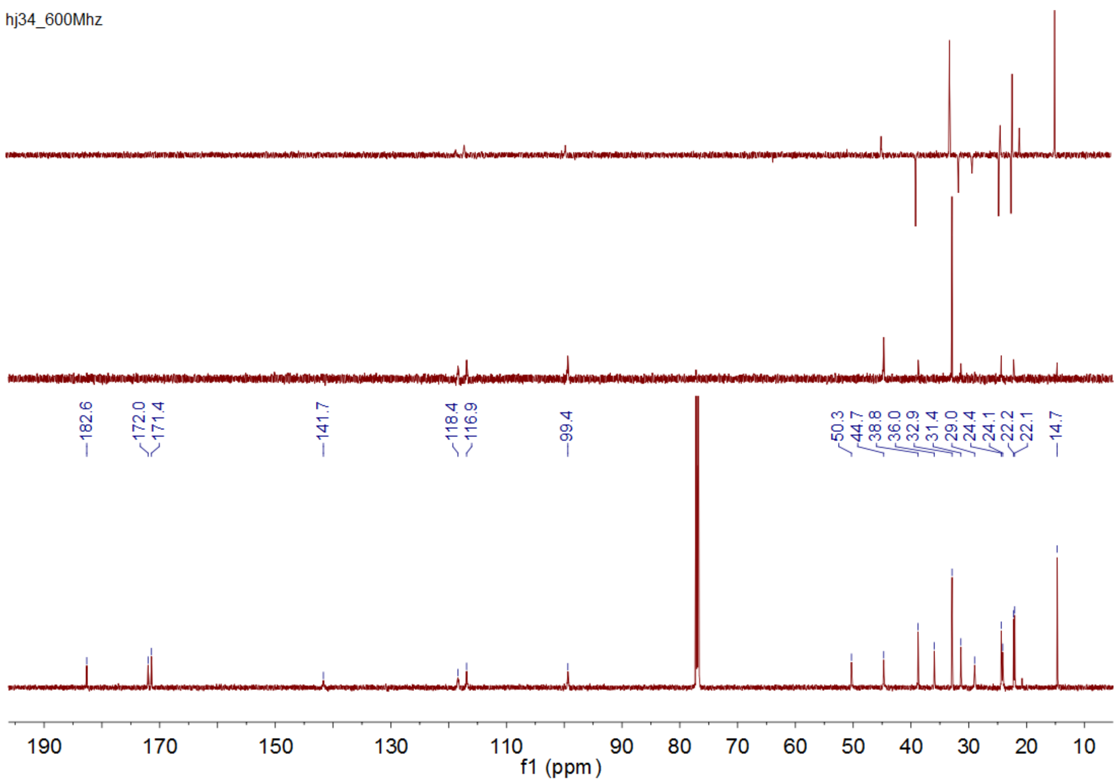

Figure S4. ^{13}C NMR spectrum of Vitenegu acid recorded in $CDCl_3$ at 125 MHz.

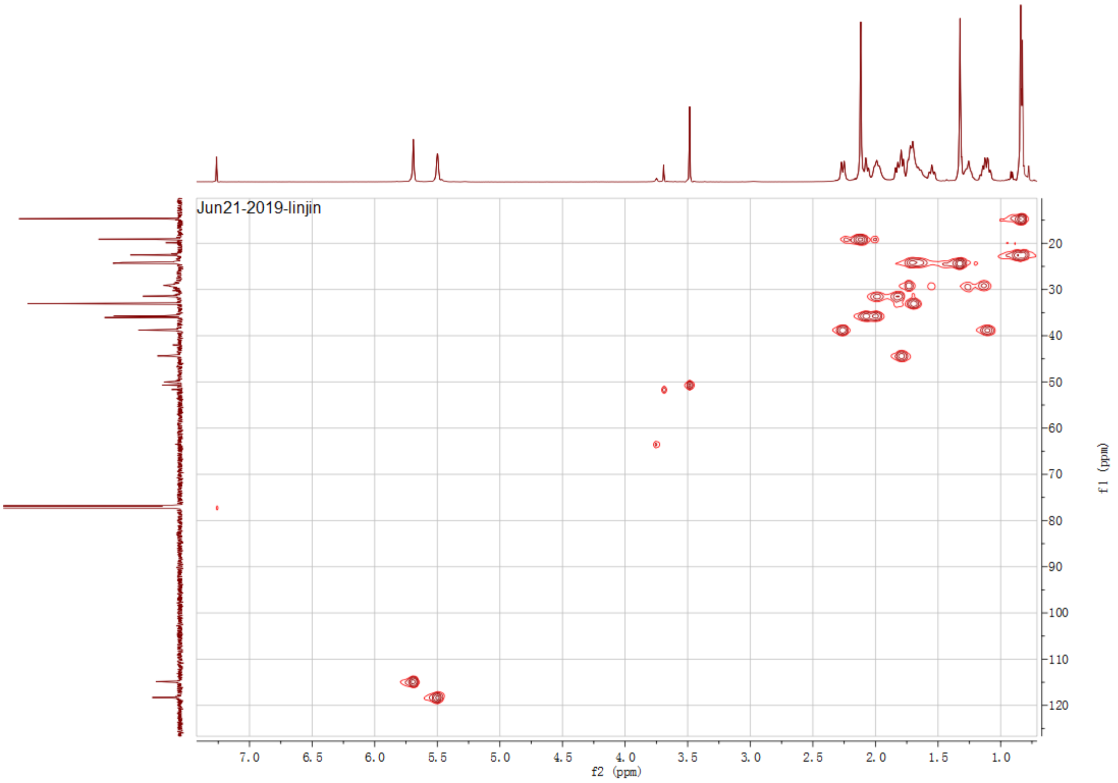

Figure S5. HMQC spectrum of Vitenegu acid recorded in $CDCl_3$.

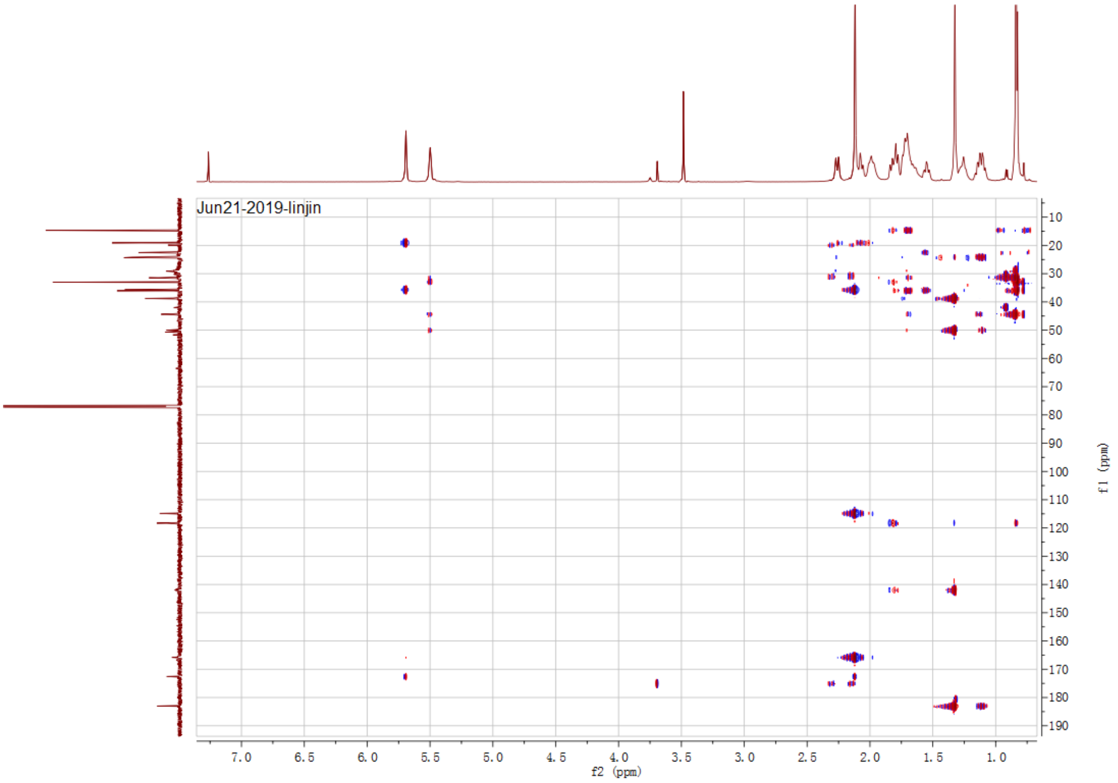

Figure S6. HMBC spectrum of Vitenegu acid recorded in $CDCl_3$.

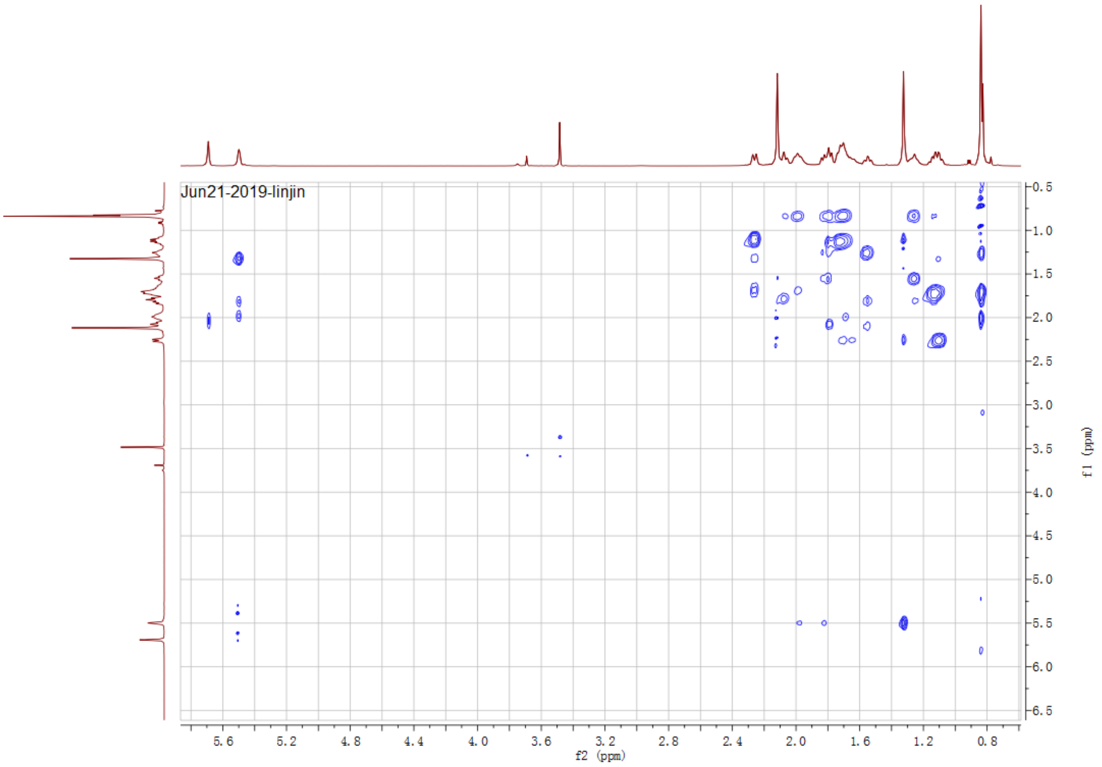

Figure S7. ROESY spectrum of Vitenegu acid recorded in $CDCl_3$.

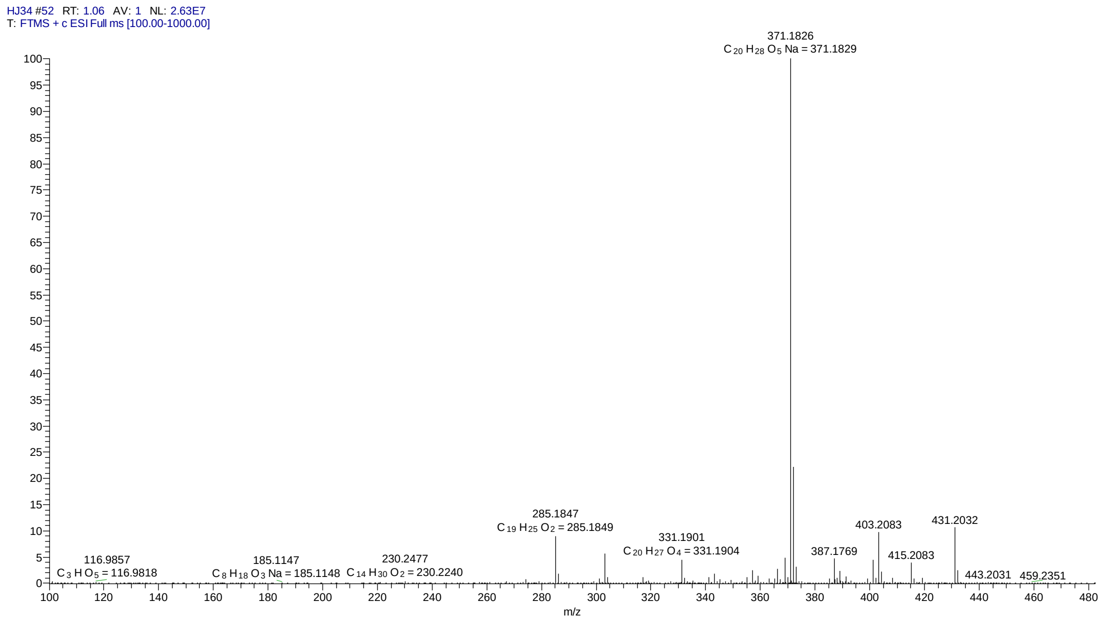

Figure S8. Mass spectrum of Vitenegu acid.

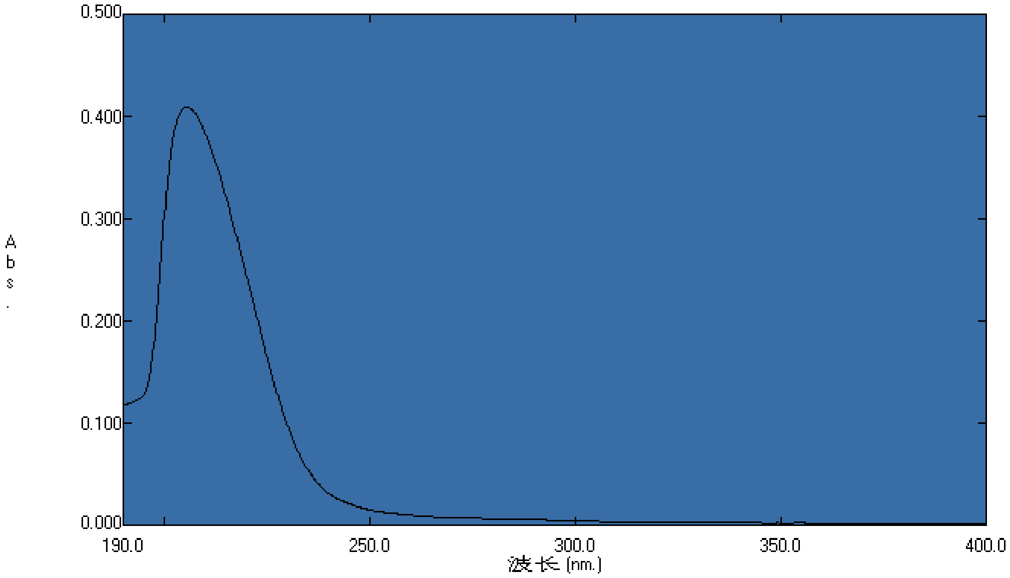

Figure S9. UV spectrum of Vitenegu acid.

Rudolph Research Analytical

This sample was measured on an Autopol VI, Serial #91058
Manufactured by Rudolph Research Analytical, Hackettstown, NJ, USA.

Measurement Date : Monday, 04-NOV-2019

Set Temperature : OFF

Time Delay : Disabled

Delay between Measurement : Disabled

n	Average	Std.Dev.	% RSD	Maximum	Minimum
5	-89.71	0.24	-0.26	-89.36	-90.00

S.No	Sample ID	Time	Result	Scale	OR °Arc	WLG.nm	Lg.mm	Conc.g/100ml	Temp.
1	HJ34	02:20:27 PM	-89.82	SR	-0.0988	589	100.00	0.110	21.5
2	HJ34	02:20:36 PM	-89.73	SR	-0.0987	589	100.00	0.110	21.5
3	HJ34	02:20:43 PM	-90.00	SR	-0.0990	589	100.00	0.110	21.5
4	HJ34	02:20:52 PM	-89.64	SR	-0.0986	589	100.00	0.110	21.5
5	HJ34	02:21:00 PM	-89.36	SR	-0.0983	589	100.00	0.110	21.5

Figure S10. Optical rotation of Vitenegu acid.

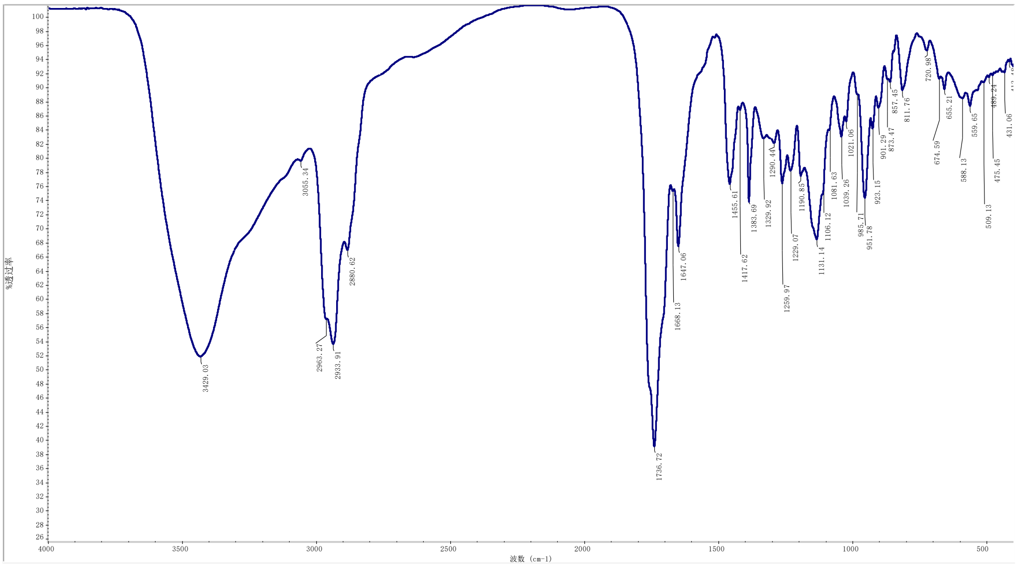

Sample Name: hj34
KBr压片
采集时间: 星期二 11月 12 14:10:12 2019 (GMT+08:00)
仪器型号: NICOLET iS10
Software version: OMNIC 9.8.372

样品扫描次数: 16
背景扫描次数: 16
分辨率: 4.000
采样增益: 1.0
动镜速度: 0.4747
光阑: 80.00

Figure S11. IR spectrum of Vitenegu acid.

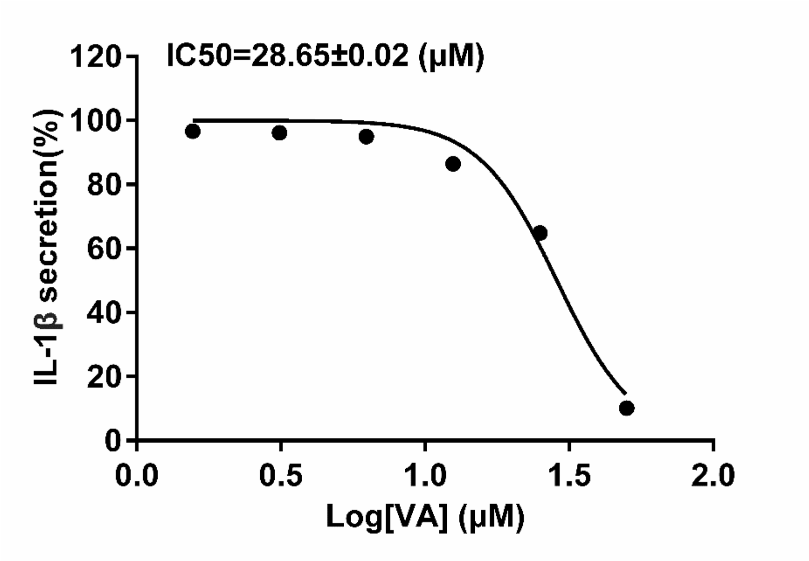

Figure S12. Production of IL-1β from mouse peritoneal macrophages pretreated with Vitenegu acid and stimulated with LPS and nigericin as measured by ELISA. Cytokine level is normalized to that of DMSO-treated control cells.

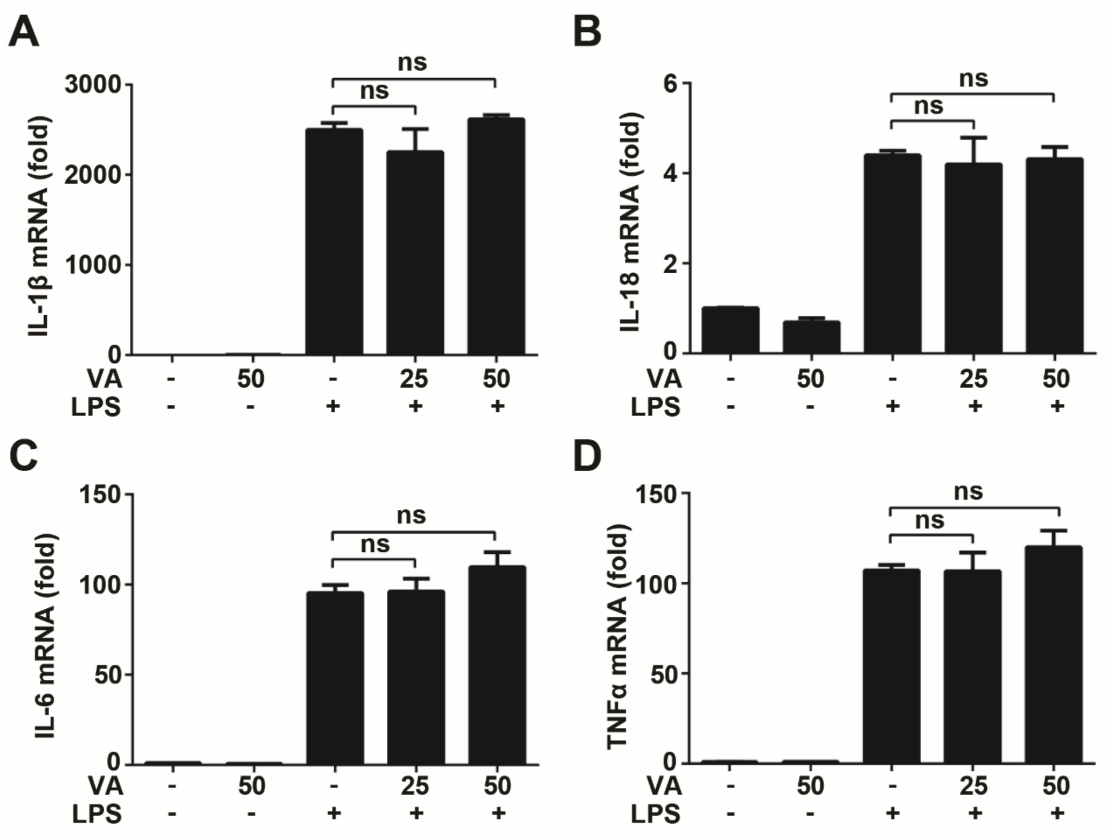

Figure S13. Mouse peritoneal macrophages were pretreated with different concentrations of Vitenegu acid (0, 25, 50 μM) for 2 h, stimulated with LPS for 4 h. Q-PCR analysis the expression of IL-1β (A), IL-18 (B), IL-6 (C)and TNFα (D). ns, not significant.

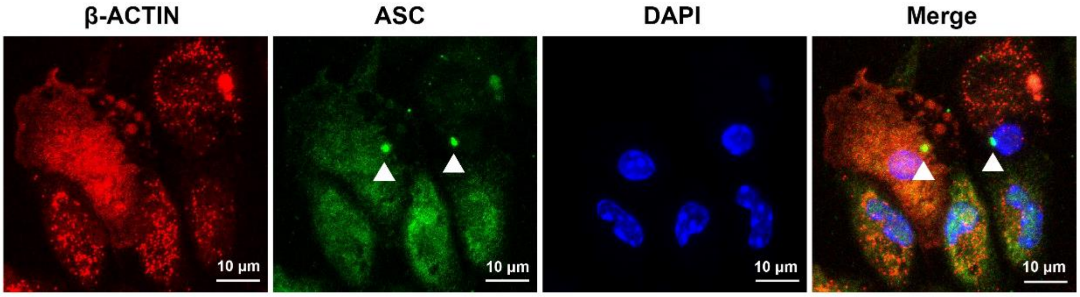

Figure S14. LPS and nigericin stimulated mouse peritoneal macrophages were stained with β-ACTIN, ASC and DAPI (original magnification 630×, scale bar 10 μm).

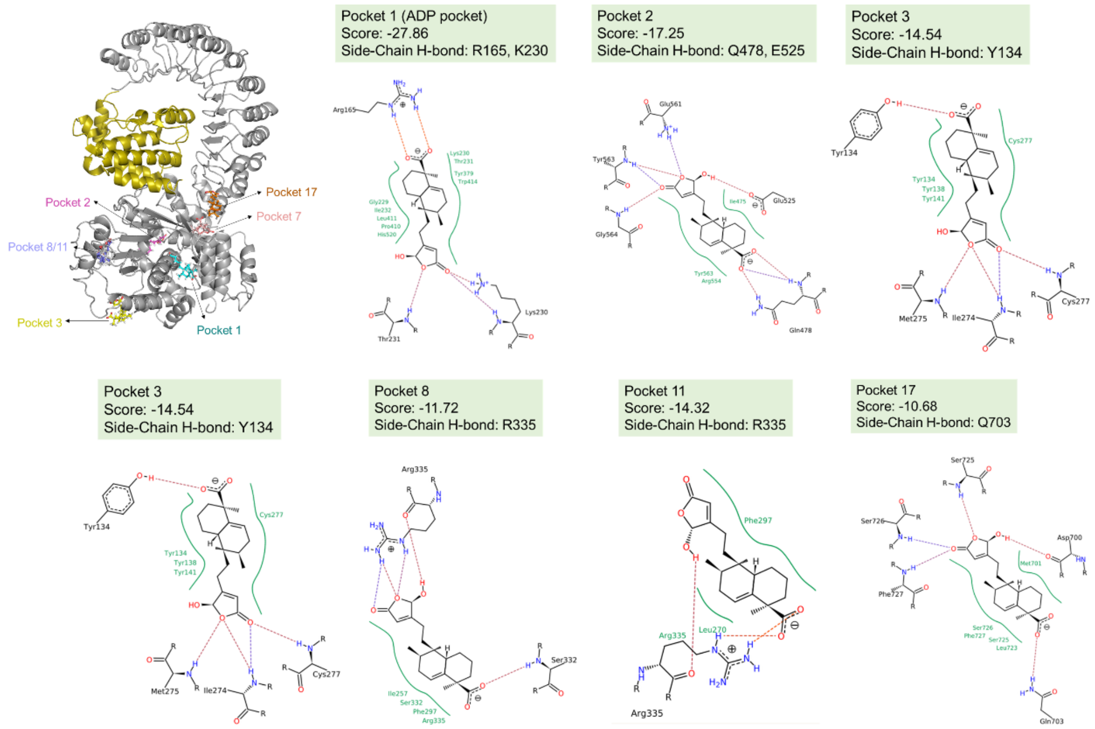

Figure S15. Top-scored binding sites on NLRP3.

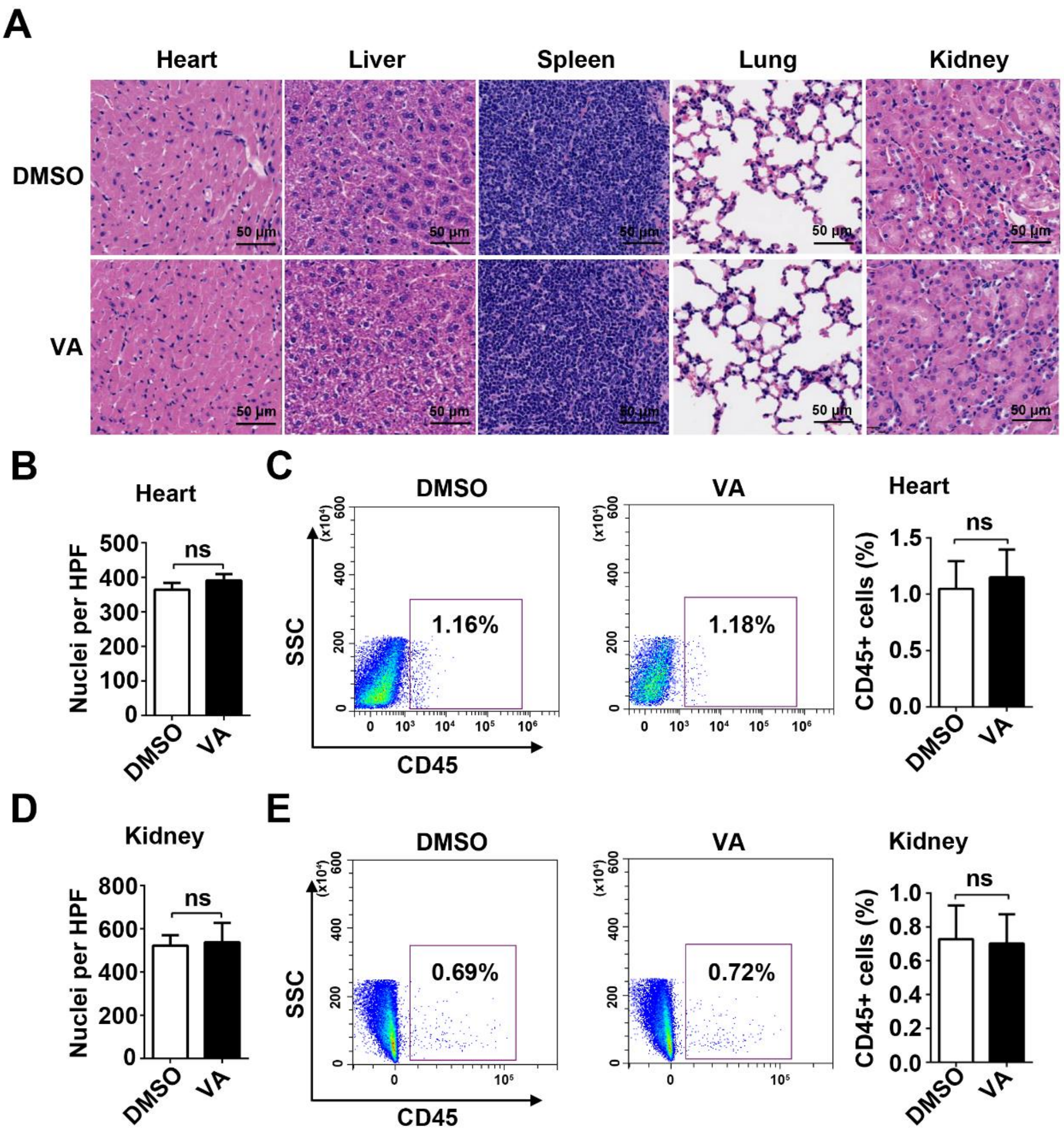

Figure S16. (A) H&E staining of major organs (heart, liver, spleen, lung and kidney) obtained from mice after treatment with DMSO or Vitenegu acid (10 mg/kg) for 6 h. Images were taken by microscope. The original magnification was 400 ×. Scale bar, 50 μm. (B) The number of nuclei per HPF (high power field) in the heart of mice treated with DMSO or Vitenegu acid. (C) Flow cytometry analysis of $CD45^+$ cells in the heart of mice treated with DMSO or Vitenegu acid. (D) The number of nuclei per HPF in the kidney of mice treated with DMSO or Vitenegu acid. (E) Flow cytometry analysis of $CD45^+$ cells in the kidney of mice treated with DMSO or Vitenegu acid.
